# P-2036. A Quality Improvement Project to Increase Pneumococcal Vaccination Rates in a Primary Care Clinic

**DOI:** 10.1093/ofid/ofaf695.2200

**Published:** 2026-01-11

**Authors:** Maria Akiki, Chebly Dagher, Tera Falcetti, Lisa DeGennaro, Angela Stein

**Affiliations:** University of Connecticut, Hartford, CT; University of Connecticut, Hartford, CT; Saint Francis Hospital, Harftord, Connecticut; Saint Francis Hospital, Harftord, Connecticut; University of Connecticut, Hartford, CT

## Abstract

**Background:**

Despite established national guidelines, pneumococcal vaccination rates in primary care remain low. Contributing factors include limited time during outpatient visits, competing clinical demands, lack of integrated reminders, and provider uncertainty due to frequent updates in vaccine recommendations. To address these barriers, we implemented a quality improvement initiative aimed at increasing pneumococcal vaccination rates by 50% at the Gengras Adult Primary Care Clinic, Saint Francis Hospital within one year of implementing the intervention.

**Figure 1:**
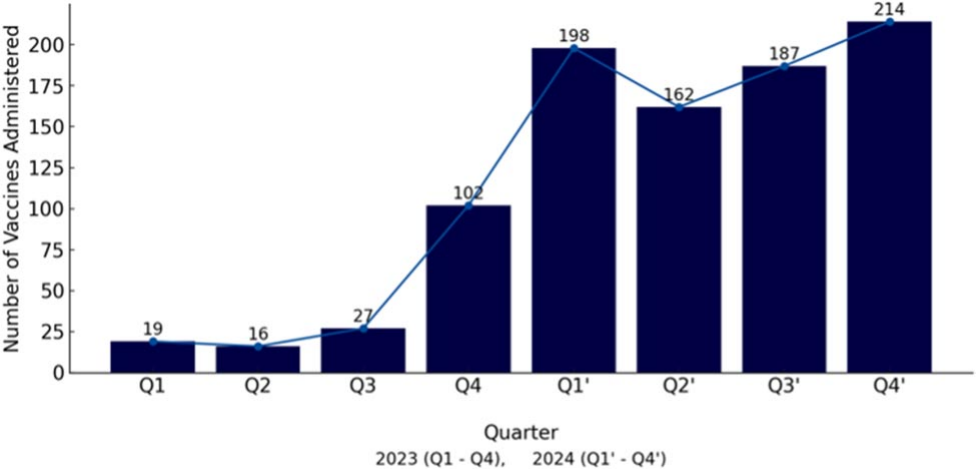
PCV20 Vaccination Trends Before and After Intervention

**Methods:**

Baseline pneumococcal vaccination performance was evaluated by reviewing the number of 20-valent pneumococcal conjugate vaccines (PCV20) administered at the the Gengras Adult Primary Care Clinic, Saint Francis Hospital during 2023, prior to the implementation of the intervention. The intervention consisted of two components. First, a SmartPhrase listing PCV20 indications aligned with CDC guidelines was incorporated into provider documentation templates and updated regularly. Second, quarterly educational sessions led by the clinic director were conducted using case-based discussions, reference reviews, and interactive question-and-answer segments to reinforce vaccination guidelines. Data were collected and analyzed on a quarterly basis.

**Results:**

During the pre-intervention period in 2023, a total of 164 PCV20 vaccinations were administered, with quarterly counts of 19 in Q1, 16 in Q2, 27 in Q3, and 102 in Q4. Following the intervention, vaccination rates increased substantially. In 2024, quarterly vaccination counts rose to 198 in Q1’, 162 in Q2’, 187 in Q3’, and 214 in Q4’, representing a 364% overall increase compared to the baseline year (Figure 1). Patient populations before and after the intervention were comparable, with no significant differences. The notably high Q4’ value may partially reflect the impact of updated CDC guidelines that broadened vaccine eligibility criteria to include additional age groups.

**Conclusion:**

The addition of a SmartPhrase to clinical documentation, along with ongoing provider education, significantly improved pneumococcal vaccination rates, resulting in sustained increases in vaccine uptake and improved adherence to vaccination guidelines.

**Disclosures:**

All Authors: No reported disclosures

